# Brucella Peptide Cross-Reactive Major Histocompatibility Complex Class I Presentation Activates SIINFEKL-Specific T Cell Receptor-Expressing T Cells

**DOI:** 10.1128/IAI.00281-18

**Published:** 2018-06-21

**Authors:** Jerome S. Harms, Mike Khan, Cherisse Hall, Gary A. Splitter, E. Jane Homan, Robert D. Bremel, Judith A. Smith

**Affiliations:** a Department of Pediatrics, School of Medicine and Public Health, University of Wisconsin—Madison, Madison, Wisconsin, USA; b Cellular and Molecular Pathology Training Program, School of Medicine and Public Health, University of Wisconsin—Madison, Madison, Wisconsin, USA; c Department of Pathobiological Sciences, School of Veterinary Medicine, University of Wisconsin—Madison, Madison, Wisconsin, USA; d ioGenetics LLC, Madison, Wisconsin, USA; Washington State University

**Keywords:** Brucella melitensis, cross-reactivity, chicken ovalbumin, major histocompatibility complex class I, MHC-I, T cell receptor

## Abstract

Brucella spp. are intracellular pathogenic bacteria remarkable in their ability to escape immune surveillance and therefore inflict a state of chronic disease within the host. To enable further immune response studies, Brucella was engineered to express the well-characterized chicken ovalbumin (OVA). Surprisingly, we found that CD8 T cells bearing T cell receptors (TCR) nominally specific for the OVA peptide SIINFEKL (OT-1) reacted to parental Brucella-infected targets as well as OVA-expressing Brucella variants in cytotoxicity assays. Furthermore, splenocytes from Brucella-immunized mice produced gamma interferon (IFN-γ) and exhibited cytotoxicity in response to SIINFEKL-pulsed target cells.To determine if the SIINFEKL-reactive OT-1 TCR could be cross-reacting to Brucella peptides, we searched the Brucella proteome using an algorithm to generate a list of near-neighbor nonamer peptides that would bind to H2K^b^. Selecting five Brucella peptide candidates, along with controls, we verified that several of these peptides mimicked SIINFEKL, resulting in T cell activation through the “SIINFEKL-specific” TCR. Activation was dependent on peptide concentration as well as sequence. Our results underscore the complexity and ubiquity of cross-reactivity in T cell recognition. This cross-reactivity may enable microbes such as Brucella to escape immune surveillance by presenting peptides similar to those of the host and may also lead to the activation of autoreactive T cells.

## INTRODUCTION

Brucellosis is a zoonotic disease caused by the Gram-negative, facultative coccobacillus bacteria of the genus Brucella. Brucella spp. reside intracellularly within the host organism, preferring macrophages and macrophage-related cells. However, they also can persist extracellularly or outside the host. Symptoms of the disease are variable, including undulant fever and osteoarticular, genitourinary, and neurological complications. Within the host, Brucella has demonstrated the ability either to hide from or misdirect the immune response, leading to chronic disease and complicating vaccine development (1). Although cytotoxic T lymphocytes (CTL) are a potentially major contributor to the control of brucellosis (2–4), the actual role of major histocompatibility complex class I (MHC-I)-restricted CTL is unclear. One study demonstrated that the absence of perforin did not affect the level of infection (5, 6). On the other hand, in a study by Oliveira et al., β2m^−/−^ mice were impaired in containment of Brucella infection (7), and Murphy et al. showed that CD8 T cell depletion exacerbated disease (8). Brucella has the ability to sabotage adaptive immune response through undefined suppressive or regulatory means, leading to the appearance of apparently exhausted CD8 T cells (3). The events producing exhaustion, as well as the nature of this state during chronic Brucella infection, await better definition but nevertheless suggest that CTL could be key in limiting infection if not suppressed. In other model systems of CD8 exhaustion, notably lymphocytic choriomeningitis virus (LCMV), the study of T cell responses has benefited tremendously from the availability of specific research tools such as T cell receptor (TCR) transgenics. In comparison, Brucella research has been relatively hindered by the inability to identify antigen-specific T cells. Although peptide epitopes have been published, there are no functional tetramers. To address this deficit, we sought to engineer Brucella to express a defined antigen that the infected antigen-presenting cell (APC) would present in the context of MHC-I to more readily characterize the immune response to Brucella infection using a mouse model.

Due to its long history in immunological research, chicken ovalbumin (OVA) is one of the best-characterized model antigens, with epitopes that have been mapped for several mouse strains. Transgenic mice expressing the variable region of the TCR specific to the OVA peptide SIINFEKL (9) are referred to as OT-1. Every CD8^+^ T cell expresses this TCR transgene (10). The combination of OT-1/TCR-transgenic T cells and the OVA-derived peptide SIINFEKL in the context of H2K^b^ is the most widely examined TCR-peptide-MHC (TCR-pMHC) complex (10, 11). Because of these readily available research tools, OVA has been a reference protein used to study CD8 T cell responses in other intracellular infections. Previous research has shown that intracellular bacteria such as Listeria monocytogenes and Mycobacterium bovis BCG expressing the OVA antigen induce strong antigen-specific primary and memory CD8 T cell responses (12–15).

In this study, we engineered and characterized OVA-expressing Brucella with the intent of studying primary and secondary CD8 T cell responses in acute and chronic brucellosis using the mouse model. Unexpectedly, we found that the research tools used to analyze OVA antigen, specifically, the cloned OT-1 TCR that recognizes the SIINFEKL peptide presented by H2K^b^, reacted to native Brucella infection as well. We therefore hypothesized that the Brucella proteome contains sequences similar to, or mimicking, the OVA SIINFEKL peptide. These results suggest that the OT-1 TCR transgenic mice may be used to study native Brucella infections and further raise questions about the nature of cross presentation and molecular mimicry.

## RESULTS

### Engineering and characterization of OVA antigen-expressing Brucella.

**FIG 1:**
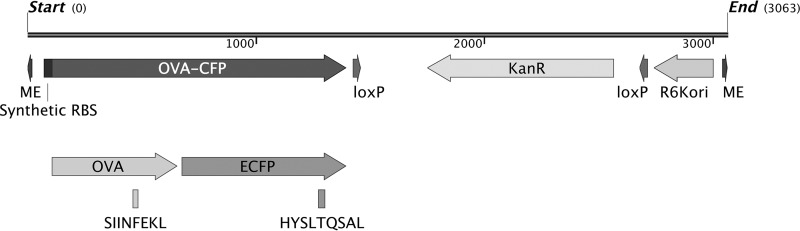
Transposon map of inserted elements. OVA-CFP and kanamycin coding sequences and the synthetic RBS and R6K origin of replication for rescue cloning are displayed. Also represented are the locations of the SIINFEKL peptide in the partial chicken ovalbumin sequence and the HYSLTQSAL peptide in the ECFP sequence. ME (mosaic ends) and loxP sites for Cre/lox recombination are also displayed.

**FIG 2:**
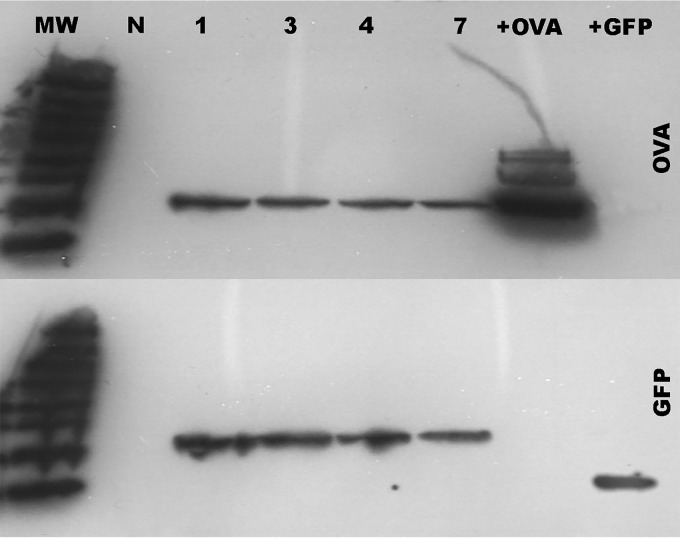
Western analyses of transposon-transformed Brucella lysates. Equivalent amounts of Brucella clones were lysed, denatured in SDS-Laemmli sample buffer, PAGE separated, and blotted. Antibodies to chicken ovalbumin (OVA) or green fluorescent protein (GFP) were used along with horseradish peroxidase (HRP) secondary antibody. Chemiluminescence was detected by X-ray film. Lanes: N, parental Brucella; 1, 3, 4, and 7, transposon-transformed Brucella clones; +OVA, chicken ovalbumin; +GFP, green fluorescent protein; MW, molecular weight marker.

**FIG 3:**
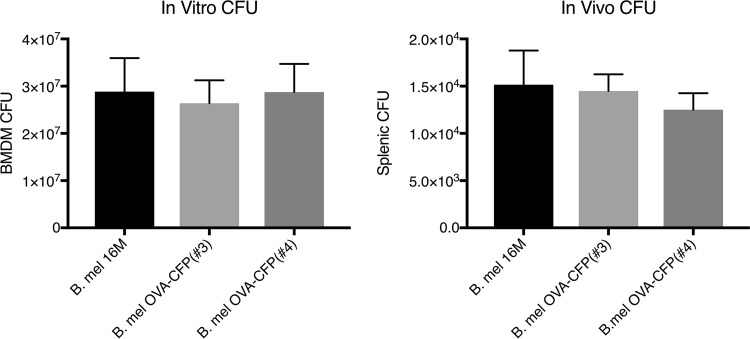
*In vitro* and *in vivo* CFU of Brucella-infected macrophages and splenocytes. Parental Brucella cells along with transformed Brucella clones 3 and 4 were used to infect C57BL/6 bone marrow-derived macrophages (BMDM) in culture (MOI, 100) for 24 h or to infect mice (C57BL/6) at 2 × 10^6^ bacteria for 7 days. Data are representative of three experiments.

Our long-term objective being acute and chronic brucellosis immunological studies, we engineered Brucella to express well-characterized antigens with readily available antigen-specific research tools. Brucella melitensis 16M was transformed to express a fusion protein consisting of a fragment of chicken ovalbumin (OVA) and cyan fluorescent protein (CFP). This fusion protein sequence was determined to have a predicted probability of antigenicity of 0.9 as measured by ANTIGENpro software using the scratch protein predictor (http://www.ics.uci.edu/~baldig/scratch/index.html). The nucleic acid sequence contains a ribosome binding sequence (RBS) optimized for Brucella, and the promoter would be provided by the insertion gene (Fig. 1). The OVA sequence selected contained OVA residues 257 to 264 (OVA_257–264_; SIINFEKL), the well-characterized H2K^b^-restricted peptide epitope (16), and the CFP portion contained the H2K^d^-restricted epitope HYLSTQSAL (17). A library of Brucella transposon transformants was made, and rescue cloning was performed to determine the transposon insertion site (Table 1). Western blotting of chosen clones using anti-OVA or anti-green fluorescent protein (anti-GFP)-specific antibodies determined protein expression (Fig. 2). The viability of transformed clones was compared to that of parental Brucella by growth in broth as well as intracellular growth *in vitro* and *in vivo* by measuring CFU from infected bone marrow-derived macrophages (BMDM) in culture or CFU from splenocytes of infected mice (Fig. 3). Clones with growth comparable to that of the wild type (clones 3 and 4) with insertions in *BMEI 1025* and *BMEII 0058*, respectively (Table 1), were chosen for further evaluation.

**TABLE 1:** Insertion genes^*a*^

Annotation	Gene	Clone no.
BMEI 0038	Hypothetical protein	16
BMEI 0039	Acetyl-coenzyme A carboxylase carboxyl	27
BMEI 0063	Membrane-spanning protein	22
BMEI 0799	Methylmalonyl-CoA mutase	14
BMEI 1025	Outer membrane protein e	1, 2, 3
BMEI 1203	Ribonuclease d	23, 28
BMEI 1496	tRNA (uracil-5)-methyltransferase	10
BMEI 1610	Cytosolic protein	15
BMEI 1611	Dihydroorotate dehydrogenase	20
BMEI 1848	Dihydroxy-acid dehydratase	5
BMEI 1849	Thiol:disulfide interchange protein cycy precursor	19
BMEII 0058	Hypothetical protein	4, 7, 8
BMEII 0135	5-carboxymethyl-2-hydroxymuconate semialdehyde	6, 9
BMEII 0136	Homoprotocatehuate 2,3-dioxygenase	25, 26
BMEII 0477	Urinate isomerase	11, 13, 17
BMEII 0478	D-mannonate oxidoreductase	6, 12, 19
BMEII 0681	Virulence protein	21
BMEII 0171	Cytosolic protein	18
BMEII 0798	Hypothetical protein	24

a Clones 3 and 4 were used for further functional assays.

### OVA_257–264_ (SIINFEKL) is presented by H2K^b^ in OVA-expressing Brucella-infected mouse BMDM.

**FIG 4:**
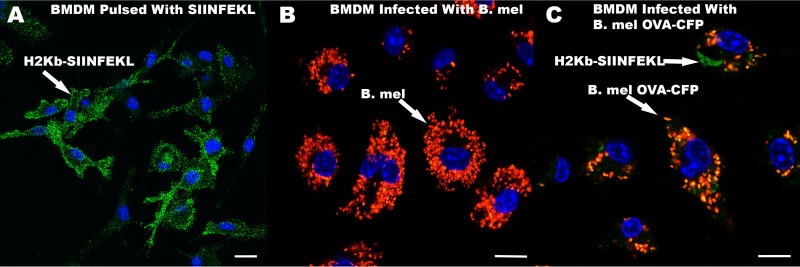
Fluorescence microscopy analyses of H2K^b^-SIINFEKL. BMDM from C57BL/6 mice were either pulsed with the SIINFEKL peptide (A), infected with parental B. melitensis (B. mel) at an MOI of 1,000 (B), or infected with B. melitensis OVA-CFP (clone 4C) at an MOI of 1,000 (C). The two B. melitensis strains expressed tdTomato (Red). Cells were fixed and stained with H2K^b^-SIINFEKL antibody (green). SIINFEKL-pulsed cells indicated that H2K^b^ was bound to SIINFEKL as expected. Bars, 10 μm.

The next objective was to determine whether the OVA-GFP fusion protein expressed by the Brucella transformants could be processed and presented by host cell MHC-I. More specifically, to determine if the OVA SIINFEKL peptide would be processed and presented on cell surface MHC-I, we employed an antibody specific for H2K^b^ bound to the SIINFEKL peptide. BMDM from C57BL/6 mice were infected with OVA-expressing Brucella and analyzed by fluorescence microscopy (Fig. 4). Results indicate that SIINFEKL peptide-MHC-I complexes could be directly visualized on the Brucella OVA-infected macrophages.

### Antigen from wild-type Brucella mediates OT-1 (OVA-specific) T cell activation and generates effectors capable of recognizing SIINFEKL-bound MHC-I.

**FIG 5:**
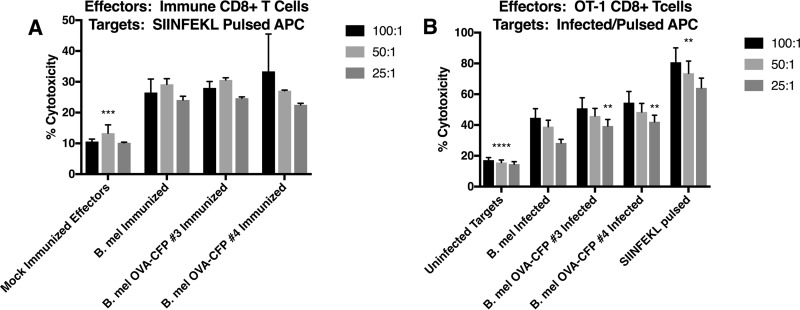
Cytotoxicity assays. (A) C57BL/6 mice were immunized with parental B. melitensis or OVA-expressing variants. Effector splenic CD8^+^ cells from these mice were used against SIINFEKL-pulsed splenocyte targets at the indicated effector-to-target ratio (E/T). Nonspecific cell death in unpulsed targets was subtracted to yield SIINFEKL-specific cytotoxicity. (B) OT-1 CD8^+^ effectors were used against parental *B. melitensis*- or OVA-expressing variant-infected target DC2.4 dendritic cells. SIINFEKL-pulsed targets were used as a positive control. Data are representative of four independent experiments. **, *P* < 0.05 (SIINFEKL-pulsed targets were significantly different from B. melitensis and variants at all E/T ratios). Additionally, **, *P* < 0.05 (clones 3 and 4 were significantly different from parental B. melitensis at an E/T ratio of 25:1). ***, *P* < 0.001 (significantly different from immunized groups). ****, *P* < 0.0001 (significantly different from infected or peptide-pulsed targets). *P* values reflect one-way ANOVA statistical analyses.

**FIG 6:**
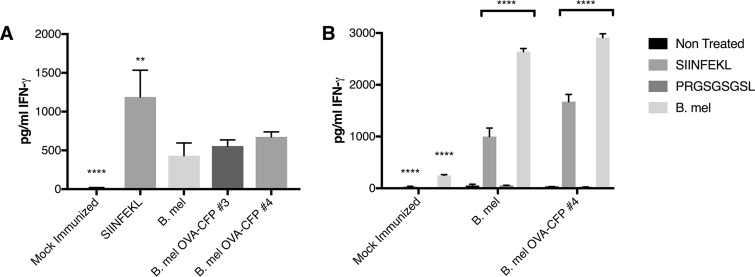
IFN-γ production by effectors from immunized mice. C57BL/6 mice were immunized with parental B. melitensis or OVA-expressing variants (clones 3 and 4) or with the SIINFEKL peptide in adjuvant as a positive control. (A) Splenocytes were pulsed with SIINFEKL peptide and assayed for IFN-γ production by ELISA. (B) Splenocytes from a separate experiment were either not pulsed (Non Treated) or pulsed with SIINFEKL or PRGSGSGSL (a random, negative-control peptide) or infected with B. melitensis (MOI, 100). **, *P* < 0.05 (statistically different from B. melitensis and variants); ****, *P* < 0.0001 (statistically different from peptide-immunized or infected splenocytes). One-way ANOVA statistical analyses were used.

As these results indicated that the OVA-CFP fusion protein was processed in infected cells and that SIINFEKL was presented by H2K^b^, we proceeded to T cell immune response studies. Unexpectedly, CD8^+^ effector T cells from mice immunized with parental and OVA-expressing Brucella strains both reacted to SIINFEKL-pulsed targets (Fig. 5A). Indeed, effectors from control B. melitensis-immunized animals lysed OVA peptide-pulsed targets at a level not significantly different from that observed with the OVA-expressing Brucella. These data indicate that B. melitensis immune cells can recognize OVA peptide-MHC-I. Similar results were observed in separate experiments examining gamma interferon (IFN-γ) cytokine production. B. melitensis-immunized effectors produced IFN-γ in response to the SIINFEKL peptide at levels not significantly different from those of the B. melitensis OVA-immunized effectors (Fig. 6A). As expected, IFN-γ expression from Brucella-immunized animals was significantly less than from OVA peptide-immunized animals. To confirm that the immunized cell response to SIINFEKL was specific, a separate immunization experiment was performed using SIINFEKL and a scrambled peptide to pulse the cells (Fig. 6B). Production of IFN-γ by splenocytes from Brucella-immunized mice was also significantly higher in the presence of the SIINFEKL peptide than in the presence of a scrambled control peptide.

To affirm these unexpected results, we altered our CTL assay approach by utilizing the well characterized SIINFEKL-specific T cell receptor-expressing OT-1 CD8^+^ T cells from TCR transgenic mice. This time, the effectors (OT-1) were OVA peptide specific and the targets were Brucella infected. Targets pulsed with SIINFEKL served as a positive control. Again, non-OVA-expressing Brucella-infected targets were lysed by OT-1 effectors at levels similar to those of OVA-expressing Brucella (Fig. 5B). As expected, OVA peptide-pulsed targets lysed at significantly higher levels.

**FIG 7:**
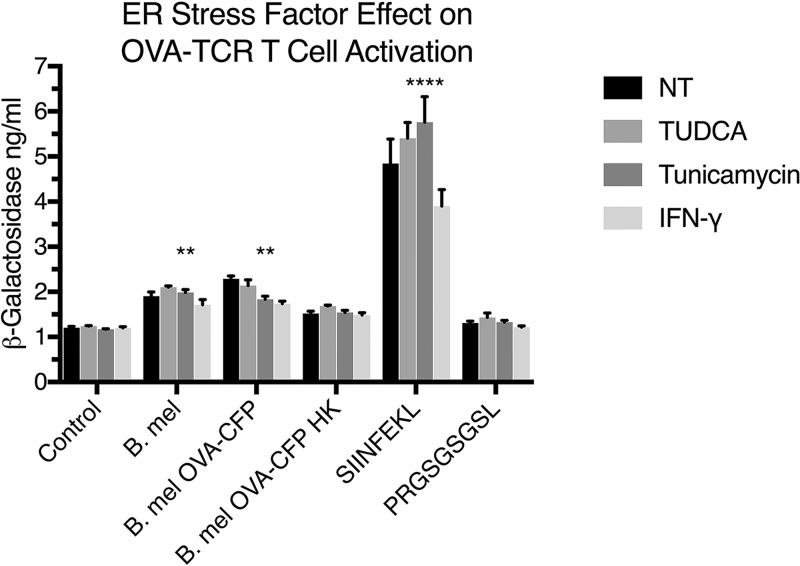
ER stress and IFN-γ effects on B3Z T cell hybrid activation by infected or peptide-pulsed APCs. DC2.4 dendritic cells were not treated (NT) or treated with UPR inhibitor TUDCA (500 ng/ml), UPR inducer tunicamycin (50 ng/ml), or IFN-γ (10 ng/ml) for 24 h. APCs were subsequently infected (MOI, 100) with Brucella melitensis (B. mel), OVA-expressing B. melitensis (B. mel OVA-CFP), or heat-killed OVA-expressing B. melitensis (B. mel OVA-CFP HK) or pulsed (10 μg/ml) with peptide for 24 h. Control DC2.4 cells were not infected or pulsed with peptide (SIINFEKL or scrambled negative control). B3Z T cell hybrids were added, and levels of TCR activation were measured through ONPG assay. Medians and standard deviations from three experiments are shown. Statistical differences from control and scrambled peptide using one-way ANOVA statistical analyses: **, *P* < 0.05; ****, *P* < 0.0001.

One possible explanation of the results presented above could be that Brucella infection nonspecifically activates T cells because of its effect on antigen-presenting cells or that we may be observing cross-reactivity of the OT-1 receptor to mouse peptides. Brucella infection induces endoplasmic reticulum (ER) stress and IFN production, either of which could potentially modulate antigen presentation (1, 18, 19). The SIINFEKL-specific responses by Brucella-immune cells were significantly greater than in unpulsed or scrambled controls (Fig. 6B), arguing against nonspecific host stimulation of T cells as the sole explanation. However, to address this theory more directly, we treated the antigen-presenting cells with infection-associated factors that could potentially alter MHC-I-peptide presentation. The drugs tauroursodeoxycholic acid (TUDCA) and tunicamycin inhibit and enhance, respectively, the unfolded protein response (UPR) (20). IFN-γ is known to enhance MHC-I expression (21). To further isolate the effects on antigen presentation and simplify responder population, we utilized the B3Z CD8^+^ T cell hybridoma with a TCR specific for the OVA (SIINFEKL)-H2K^b^ complex. The cell line was transfected with a *lacZ* reporter gene driven by the nuclear factor of activated T cells (NFAT) element of the human interleukin-2 (IL-2) enhancer. The H2K^b^ presentation of SIINFEKL to B3Z cells activates NFAT and results in β-galactosidase synthesis, which can be detected as blue staining of cells visualized by microscopy or quantitated by development of the ONPG (*o*-nitrophenyl-β-d-galactopyranoside) chromogenic substrate. The B3Z reporter system is widely used in T cell activation studies (22). Results shown in Fig. 7 indicate that ER stress modulation or IFN-γ treatment did not result in further activation of B3Z reporter T cells bearing the OVA-specific TCR, by SIINFEKL peptide or whole-Brucella infection.

### Detection of near-neighbor T cell-exposed motifs (TCEM) in Brucella indicates possible molecular mimicry.

These surprising results led us to theorize that there might be cross-reactivity of the OVA-reactive T cells to structurally related peptides derived from Brucella. We performed a search of the B. melitensis proteome for the SIINFEKL sequence. The proteome of B. melitensis does not contain a peptide identical to SIINFEKL. However, 38 peptides that comprise a P4 to P8 T cell-exposed motif matching one of the peptides having near-neighbor physicochemical characteristics were found. Table S1 in the supplemental material shows these peptides, the Brucella proteins of origin, and the predicted binding affinity to murine MHC-I alleles H2K^b^ and H2D^b^ of the nonamer peptides that contain these motifs. For those peptides having the highest predicted binding affinity to H2K^b^ or H2D^b^, the probability of C-terminal cathepsin excision was examined. Interestingly, for peptides that were predicted to have a high probability of C-terminal cleavage by either cathepsin S or L to permit MHC-I binding, cleavage probability was highest at position P10, i.e., yielding a decamer peptide. This is consistent with prior observations that indicate that a decamer may be more likely to be initially excised than a nonamer (23). This selection process yielded a ranking of peptides for further study, among which four were selected for testing. In making this selection, we also considered proteins that had clearly detectable expression observed during our previous proteomics and transcriptome sequencing (RNA-seq) studies of infected cells (18). For controls, we selected a peptide comprising a near-neighbor motif that was predicted to have low affinity for H2K^b^ or H2D^b^ and a random nonamer peptide. Table 2 describes the peptides used in these studies.

**TABLE 2:** Peptides used for cross-reactivity studies

UniProt^*a*^	Curation	9-Mer	H2K^b^	H2D^b^	TCEM^*c*^	Prot^*d*^	RNA-seq^*e*^
P65217	Guanylate kinase	KSIINAERL	−1.17^*b*^	−2.56	xxxINAERx	906	1,210
Q8YEB3	Translation initiation factor 2	KNKINLDKL	−0.94	−1.46	xxxINADKx	51	239
Q8YCC4	Aldehyde dehydrogenase A	SSSIQFEKV	−1.35	−1.59	xxxIQFEKx	113	114
Q8YGJ1	50s ribosomal protein L13 rplM	VIIINADKV	−1.84	−2.07	xxxINADKx	664	2,035
Q8YDJ8	Zinc ABC transporter	PQKINIDRT^*f*^	0.24	0.38	xxxINIDRx	802	1,251
NA^*g*^	Randomly generated	PRGSGSGSL^*h*^			xxxSGSGSx	NA	NA
P01012	Chicken ovalbumin	SIINFEKL^*i*^			xxINFEKx	NA	NA

a UniProt designation (www.uniprot.org).

b A more negative number indicates higher binding affinity for K^b^ or D^b^.

c TCEM, T cell-exposed motif.

d Extracellular Brucella Proteomics abundance rank.

e Intracellular RNA-seq abundance rank.

f Near-neighbor peptide with predicted low binding affinity for K^b^ and D^b^.

g NA, not applicable.

h Scrambled 9-mer negative control.

i 8-mer positive control.

Examination of the proteomes of 4 or 5 distinct isolates each of 140 other bacterial pathogens, from 14 genera, identified that peptides comprising the P4 to P8 motifs of near neighbors of SIINFEKL are not uncommon. The frequencies of such near-neighbor peptides in other pathogens are shown in Table S2 in the supplemental material. Each bacterial proteome examined contains from 4 to 45 near-neighbor motifs of SIINFEKL that may produce cross-reactions similar to those shown here for B. melitensis, if the flanking amino acids in each context are conducive to cathepsin cleavage and to MHC-I binding. A similar incidence of the near-neighbor peptides was detected in the proteomes of 20 bacteria found in the gastrointestinal microbiome (data not shown).

### Putative Brucella peptides can activate OVA-specific TCR-bearing T cells.

**FIG 8:**
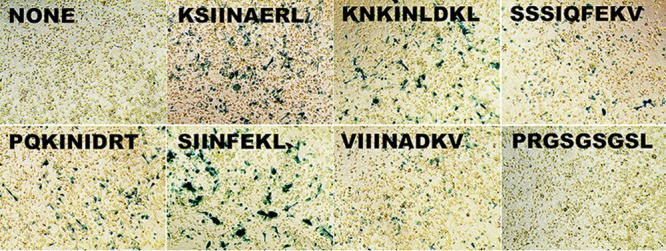
B3Z lacZ expression of T cell activation. DC2.4 dendritic cells were pulsed with 10 μg/ml of the indicated peptides and mixed with B3Z T cell hybrids. PRGSGSGSL is the scrambled peptide control. After 24 h, T cell activation was monitored by β-galactosidase expression. Blue cells indicate peptide-MHC-I binding of the TCR and T cell activation.

**FIG 9:**
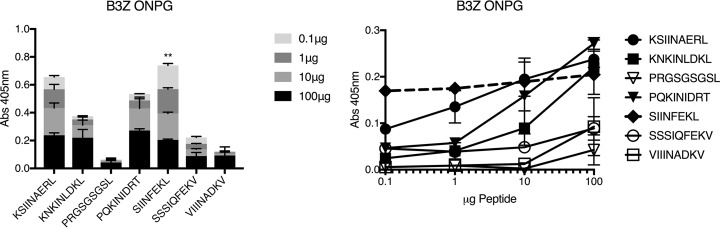
T cell activation by peptide-MHC-I at different peptide dilutions. DC2.4 dendritic cells were pulsed with indicated amounts of peptide and mixed with effector B3Z T cell hybrids. Levels of TCR activation were measured through ONPG assay. Medians and standard deviations from six assays are shown. (Left) Data are expressed as stacked columns of various dilutions of each peptide. (Right) The same data as for the left panel are expressed as a line graph. The dashed line is the SIINFEKL reference control. One-way ANOVA statistical analyses of median values indicated that SIINFEKL-treated samples were significantly different (**, *P* < 0.05) from all other treatments at only 0.1-μg peptide concentration.

To assay cross-reactivity of the putative Brucella peptides, we employed the B3Z cell line (24) and peptide-pulsed DC2.4 mouse dendritic cell line (H2K^b^) as APC. Visual scanning of the *lacZ*-stained cells revealed that all the peptides tested in Table 2, except the random sequence, had some level of staining above background (no peptide added). In fact, three of the peptides (KSIINAERL, PQKINIDRT, and KNKINLDKL) were observed to have staining similar to that of SIINFEKL (Fig. 8). We then repeated our assays using peptide dilutions to assess the avidity of the pulsed peptide-MHC-I and TCR interaction. The ONPG assays (Fig. 9) verified our lacZ results indicating that three of the experimental peptides (KSIINAERL, PQKINIDRT, and KNKINLDKL) had high levels of TCR (NFAT) activation. ONPG assays also revealed different avidities for the peptides. Figure 9B shows that whereas SIINFEKL avidity did not change much at all dilutions tested, the Brucella peptide avidity decreased with dilution. Notably, the native Brucella KSIINAERKL peptide did not differ significantly from the SIINFEKL peptide until the dilution reached 0.1 μg/ml.

**FIG 10:**
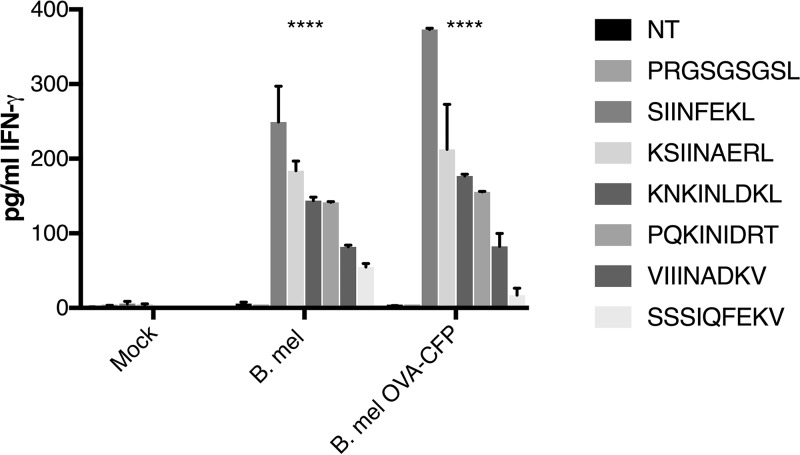
Immune response to peptides. C57/BL6 mice were immunized with 10^6^ Brucella melitensis (B. mel) cells, Brucella melitensis cells expressing OVA-CFP antigen (B. mel OVA-CFP), or diluent (Mock). After 3 weeks, splenocytes were harvested and stimulated with peptide (50 μg) for 24 h, and supernatant was assayed for IFN-γ by ELISA. Absorbance readings represent the medians and standard deviations for four mice from each immunization group. ****, *P* < 0.0001 (significantly different from mock-immunized group in two-way ANOVA statistical analyses).

To determine if infection with the parental B. melitensis 16M generated immune responses to these OVA-TCR cross-reactive peptides *in vivo*, splenocytes from mice immunized with either B. melitensis or B. melitensis OVA were assayed for IFN-γ production after stimulation with peptide (Fig. 10). Immune cells did indeed respond to the panel of cross-reactive peptides with greater cytokine production than to scrambled peptide, suggesting that these epitopes may be generated *in vivo*.

**FIG 11:**
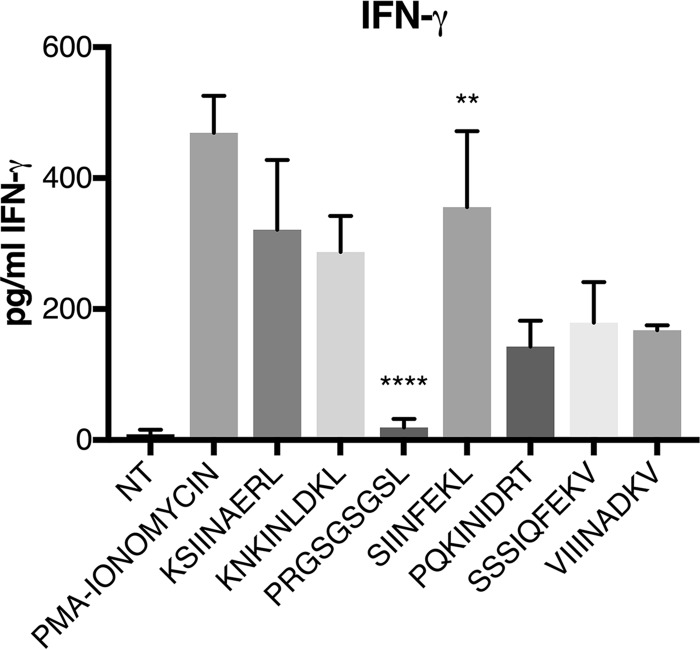
OT-1 CD8^+^ cell activation by native Brucella sequences. Splenocytes from OT-1 mice were pulsed with 50 μg of peptide for 24 h, and supernatant was assayed for IFN-γ by ELISA. Absorbance readings represent the medians and standard deviations from four experiments. NT, nontreated; **, *P* < 0.05 (SIINFEKL is significantly different from PRGSGSGSL, PQKINIDRT, SSSIQFEKV, and VIIINADKV); ****, *P* < 0.0001 (PRGSGSGSL is significantly different from all other treated samples in one-way ANOVA statistical analyses).

Finally, we determined if these native Brucella peptides could activate OVA-specific TCR using the OT-1 mouse system. Splenocytes from these mice were pulsed with the various peptides, and IFN-γ expression was measured by enzyme-linked immunosorbent assay (ELISA). Results shown in Fig. 11 confirm our findings using the B3Z β-galactosidase reporter cell line that the OT-1 TCR is cross-reactive to peptides of the Brucella proteome. Peptide immunization of the OT-1 mice was not attempted due to an anticipated hyperimmune response (9, 10). Indeed, when we tried infecting these mice with Brucella and the Brucella OVA mutants, the mice died within 6 days (data not shown).

## DISCUSSION

The immune response to Brucella melitensis is complex, as evidenced by the fact that there is currently no known effective brucellosis vaccine available. Our research goal was to engineer Brucella to express immunogenic OVA as a tool to follow antigen-specific effector and memory immune responses to Brucella infection *in vivo*, using the mouse model. Adopting this approach, we would be able to employ SIINFEKL-MHC tetramers and T cells from TCR transgenic mice. This goal was nominally fulfilled. However, the surprising result was that native Brucella also stimulated “OVA-specific” TCR of OT-1 mice. Employing a panel of near-neighbor Brucella peptides, cross-reactivity of the OVA-TCR was evident, although to a lesser extent than that of the OVA SIINFEKL that was originally used in clonal selection of the TCR (10, 11, 25). Despite the lower avidity, as indicated by the B3Z assays, Brucella peptides from infections were sufficiently immunogenic to trigger both robust cytokine and CTL responses via the OVA TCR. These results suggest that the OT-1 TCR transgenic T cells may be used to probe native Brucella immune responses as well as responses to the Brucella OVA.

A search of the Brucella melitensis proteome revealed several proteins containing T cell-exposed pentamer motif sequences with physicochemical characteristics similar to those of SIINFEKL. Such “near-neighbor” pentamers were also identified in many other bacteria. These included proteomes of Listeria monocytogenes, Salmonella enterica, and Mycobacteria bovis BCG. Listeria, Salmonella, and BCG have all been engineered to express OVA using a method similar to the approach shown here with Brucella (13, 26, 27). However, no cross-reactivity of native bacterial peptides to OVA-specific TCR was reported. This may be because the flanking amino acid context precluded binding and presentation in these bacteria or because this phenomenon has been observed with other bacteria but not reported (28). The occurrence of near-neighbor pentamers in the gastrointestinal microbiome organisms suggests that prior exposure to such peptides is difficult to avoid. Sequence scans of other Brucella species, including B. abortus, B. suis, B. neotomae, and B. ovis showed these pentamer motif sequences to be conserved. One limitation of this study is that it would not be feasible to delete all genes encoding the SIINFEKL near neighbors to definitively confirm the connection between production of SIINFEKL cross-reactive peptides and recognition of infected cells by OT-1 T cells. Additionally, the proteins containing the peptides listed in Table 2 used in these studies may be essential for survival. In searching the literature describing Brucella mutants, we could not find engineered mutants of these proteins (29–32), possibly because they did not survive the mutation process or were severely attenuated.

An intriguing alternative to our explanation could be that Brucella infection alters the presentation of the host proteome. Indeed, our previous studies have found that Brucella induces the host unfolded protein response (33, 34), and this ER stress could theoretically alter host or self-antigen presentation. Further, immune activation during Brucella infection results in IFN-γ production, which is known to upregulate MHC-I expression and self-antigen presentation. Searching the C57/BL6 mouse proteome revealed no SIINFEKL peptide sequence. However, applying our near-neighbor algorithm did reveal 22 instances of ∼∼∼INFEK∼ sequences that would potentially be exposed to the T cells. Nevertheless, the predicted binding affinity to H2K^b^ or H2D^b^ was low compared to that of the Brucella peptides used in this study (data not shown). Testing this theory using UPR inducer/inhibitors and a cytokine APC activator did not enhance stimulation of the OVA-specific TCR-expressing B3Z T cells. These results suggest that the host (mouse) does not present an OVA-like peptide due to ER stress or APC activation by IFN-γ.

Activation of the T cell through the T cell receptor by peptide-MHC-I had been thought to be peptide specific; however, our results, along with those of others (35–39), have shown that T cells can be triggered by peptides with even minimal obvious homology to the primary immunogenic peptide, or amino acid substitutions with similar charge and size. The TCR recognizes an immunogenic complex consisting of peptide bound to MHC-I with peptides of 8 to 11 amino acids in length (40). Of those amino acids, only five are exposed to the TCR (41, 42). Given the genetic combinatorial rearrangement possibilities, an estimated 10^15^ unique TCRs could be generated in the mouse (37). However, studies have shown that there are actually <10^8^ distinct TCR clones in the human naive T cell pool (43), and the number in mice is likely similar. Since 20^9^ foreign peptide nonamers can theoretically be generated, it would be mathematically impossible for the T cell pool to recognize all foreign peptides if the TCRs were monospecific (38). Therefore, the TCR must be degenerate and cross-reactive to near-neighbor motifs as demonstrated here. In fact, a TCR is estimated to react productively with 1 × 10^6^ different MHC-peptide epitopes (44). T cell cross-reactivity has also been documented using the OT-1 transgenic mouse model that we used in this study (10). Nevertheless, the degeneracy of the TCR correlated with differences in avidity to the peptide-MHC-I complexes, as others have reported (45). Our studies confirmed that higher concentrations of a degenerate peptide were needed to activate the “SIINFEKL-specific” TCR to the same level of activation as SIINFEKL itself. Noteworthy is our observation that antibody to H2K^b^-bound SIINFEKL is apparently not degenerate but SIINFEKL specific and could be visualized on Brucella-OVA-infected cells only and not on Brucella-infected cells (Fig. 4). Whether this is truly due to antibody specificity or perhaps assay sensitivity would need further investigation.

Our results underscore the complexity and ubiquity of molecular mimicry in T cell recognition. The potential for extensive sharing of nonamers between pathogens, the gastrointestinal microbiome, and the human proteome has been demonstrated, for both MHC-I and MHC-II (46). This may enable microbes to escape immune surveillance by presenting peptides similar to those of the host and may also lead to microbial exposure cross-activating autoreactive T cells. Although the association between bacterial infections and autoimmune disorders is still not fully understood (47), recent reports indicate that molecular mimicry may be responsible for activation of autoimmune diseases (48–52). Consistent with this, Brucella infections have been implicated in several autoimmune diseases (53–56). The genome of Brucella melitensis is predicted to encode 3,197 open reading frames (ORFs) distributed over two circular chromosomes (57). However, even with this level of complexity, microbe-human commonality is extremely high, with 99.7% of human proteins containing bacterial pentapeptides (46, 58). Furthermore, while this study addresses continuous pentamers that are recognized by CD8^+^ T cells, such peptides are overlaid by the discontinuous pentamers presented by MHC-II and recognized by CD4 T cells, with a similar degree of potential cross-reactivity (46). Although chicken ovalbumin is not a host protein of mouse or humans, we have demonstrated here that there is enough commonality for cross-reactivity of several putative Brucella peptides with SIINFEKL. It is possible that the cross-reactivity of transgenic OT-1 immune cells to Brucella could be used in autoimmune studies in mice.

In summary, we have generated a unique tool to dissect CD8^+^ T cell responses to Brucella infection using a widely available TCR transgenic mouse model. Further, the OT-1 mice may also be used to probe native Brucella infections. Transgenic mice carrying monoclonal T cell receptors are widely used in immunological research. The results presented here raise an important caution for the interpretation of experiments based on reactions to SIINFEKL, or any other small single peptide, unless the presence of the T cell recognition motif or potential cross-reactive near neighbors within the host, its microbiome, or an organism under study is addressed. Our results also challenge the assumption that sequence homology will predict molecular mimicry. Thus, using databases comparing sequences of “self” and pathogens will almost certainly underestimate the true contribution of molecular mimicry to pathogen-triggered autoimmunity.

## MATERIALS AND METHODS

### Mice.

C57BL/6 (Harlan) and C57BL/6-Tg(TcraTcrb)100Mjb/J (Jackson) mice were housed and cared for in AAALAC-certified facilities of the University of Wisconsin School of Veterinary Medicine. Care, handling, and experimental procedures were approved by the Institutional Animal Care and Use Committee (IACUC) with strict adherence.

### Cells and cell culture.

Brucella melitensis 16M strains and all Escherichia coli strains used in this project were cultured in brain heart infusion (BHI) broth or agar at 37°C. Mouse dendritic cell line DC 2.4 (H2K^b^), and mouse monocyte cell line LADMAC were cultured in R10 medium, consisting of RPMI supplemented with 10% fetal calf serum (FCS) and 1 mM sodium pyruvate, in a humidified 37°C incubator with 5% CO_2_. The B3Z CD8^+^ T cell hybridoma cell line, specific for the SIINFEKL (OVA_257–264_/K^b^) peptide of OVA, was a kind gift from J. D. Sauer (University of Wisconsin—Madison). B3Z cells were cultured in R10 containing 500 μg/ml G418 (Geneticin). Bone marrow-derived macrophages (BMDM) were prepared by the culturing of bone marrow cells from the tibia/fibula of mice in R10 conditioned with 20% LADMAC supernatant.

### Plasmid and transposon engineering.

We used the EZ-Tn5 (Lucigen) transposon mutagenesis system for random insertion into the Brucella genome according to the manufacturer's recommended protocol. The insert was cloned into the transposon construction vector pMOD-3 <R6Kγ*ori*/MCS> so that rescue cloning could be performed to determine the insertion site within the transformed Brucella. The partial OVA sequence was amplified from the vector pPL2erm-ActA100-B8R-OVA (kind gift from J. D. Sauer). Primers incorporated a Brucella ribosome binding site (RBS) designed using the algorithm RBS Calculator v2.0 for high-translation initiation (59, 60). The primers also contained EcoRI and BamHI restriction sites for subcloning into pECFP-N1 (Clontech) to produce an OVA-CFP fusion protein. Primers were as follows: N′-TGAAAGCAAAAGCAGAGAATTCTGGAATATTTTAATTCAGTATCAAAGAGAGGTAAACATGCAAGCCAGAGAGCTCATCA; C′-TTGAGGATCCTTCAGGCTCTCTGCTGAGGAGATGCCAGACAGA. PCR was performed using the GoTaq Flexi DNA polymerase system (Promega) with 6 mM MgCl_2_ and 55°C annealing temperature. The 632-bp product was subcloned into pECFP-N1. The fusion product was inserted into the EcoRI/XbaI site of pMOD-3. Finally, the kanamycin resistance sequence was added to the SalI site from pUC4k (Amersham) that we had modified to be flanked by loxP sites. The final product was named pMOD3-OVA-CFP. The map can be seen in Fig. S1 in the supplemental material.

### Brucella transformation and rescue cloning.

Transposons were generated by PCR according to the manufacturer's recommended protocol (Lucigen). Electrocompetent Brucella cells were prepared by growing Brucella to log phase in BHI broth. Brucella cells were pelleted and washed at least four times with ice-cold water. The electrocompetent Brucella cells (50 μl) were then electroporated with the transposon (2 μl). Then, 950 μl of BHI was immediately added to the cells, followed by overnight shaking in an incubator at 37°C. The next day, 200 μl of cells was plated on BHI agar plates containing 50 mg/liter kanamycin. Plates were cultured for 5 to 7 days at 37°C. Clones were then selected and cultured in 96-well plates as a bacterial library, and clones from the library were then propagated for rescue cloning of the transposon insertion site. Rescue cloning was performed according to the manufacturer's (Lucigen) protocol. Briefly, Brucella transformant genomic DNA was extracted using the MasterPure DNA purification kit (Epicentre), and 2 μg of DNA was digested to completion with NcoI overnight to generate a fragment with intact transposon and flanking sequences. Digested DNA was religated using a FastLink DNA ligation kit (Epicentre). Ligations were column purified and transformed into electrocompetent E. coli EC100D*pir*+ cells (Epicentre) and plated on BHI agar containing kanamycin (50 μg/ml). Kanamycin-resistant colonies were selected, the plasmid was extracted, and the site of insertion was identified by sequencing the plasmid DNA bidirectionally using outward primers forward (FSP; 5′-GCCAACGACTACGCACTAGCCAAC) and reverse (RSP; 5′-GAGCCAATATGCGAGAACACCCGAGAA). Sequencing was performed at the DNA sequencing core facility of the University of Wisconsin Biotechnology Center. Sequences were compared to the 16M genome sequence to determine the site of insertion.

### Western blotting.

Protein lysate of both bacterial and mammalian cell extracts was made using the B-PER bacterial protein extraction reagent (Thermo Fisher). Proteins were prepared for SDS-PAGE by heat denaturation in Laemmli sample buffer (Bio-Rad). Equal amounts of protein were added to wells of a 4 to 20% Tris-HCl gradient gel (Bio-Rad) along with a SuperSignal molecular weight protein ladder. Separated proteins were transferred to nitrocellulose (Bio-Rad). Western blotting was performed utilizing a Pierce Fast Western blot kit as per the manufacturer's instructions. Antibodies included mouse monoclonal anti-OVA (3G2E1D9; GenTex) and mouse monoclonal anti-GFP (B-2; SCBT) and were used at recommended dilutions. Chemiluminescent blots were visualized on X-ray film.

### Fluorescence microscopy.

BMDM were prepared from C57BL/6 mice and plated on chambered coverslips (Ibidi). Some samples were then infected (multiplicity of infection [MOI], 1,000) with Brucella expressing tdTomato fluorescent protein (Clontech) for 24 h. The high MOI was chosen to increase sensitivity, consistent with our previous studies (18). Other samples were pulsed with the SIINFEKL peptide (50 μM). Cell samples were fixed in 4% paraformaldehyde and processed for fluorescence confocal microscopy. Cells were stained with a monoclonal antibody to OVA_257–264_ (OVA residues 257 to 264; SIINFEKL) peptide bound to H2K^b^ (eBioscience) and then with goat anti-mouse IgG (H+L) Alexa Fluor 488 (Dylight; Thermo Fisher). Imaging was performed at the University of Wisconsin Optical Imaging Core using either a Nikon A1RS confocal microscope or a Leica SP8 3X STED Super-resolution microscope.

### Cytotoxic T lymphocyte assay.

For the CTL assays, assessment consisted of measuring the extracellular activity of dead-cell protease by luminescence using the CytoTox-Glo cytotoxicity assay (Promega). Effector and targets for the assay varied as described below. Immune effectors were prepared as follows. Mice (C57BL/6, female, 4 weeks old) were injected intraperitoneally (i.p.) with phosphate-buffered saline (PBS; diluent control) or 2 × 10^6^ Brucella cells in 200 μl PBS (B. melitensis, B. melitensis ova-cfp 3, B. melitensis ova-cfp 4). Each group consisted of 4 mice. After 3 weeks, mice were euthanized and splenocytes were harvested. CD8^+^ T cell effectors were isolated from splenocytes using a MACS CD8a^+^ T cell isolation kit (Miltenyi). Targets consisted of splenocytes from age-controlled mice that were pulsed with the OVA_257–264_ peptide (SIINFEKL) or not peptide pulsed. Briefly, mononuclear splenocytes were suspended in complete growth medium (R10) at 5 × 10^6^/ml. The OVA peptide (SIINFEKL; GenScript) was added at 1 μl/ml from a 200 μM stock, and cells were incubated at 37°C for 1 h. To control for nonspecific cytotoxicity, unpulsed target controls were also included in the assay and this background level was subtracted from the experimental levels. Targets, effectors, and controls were plated in triplicate in 96-well round-bottom plates. Cells were incubated for 5 h and then assayed by luminometry. Specific cytotoxicity represents SIINFEKL-pulsed target cell death minus background nonpulsed target cell death. The OT-1 effector cytotoxicity assay was prepared as follows. Splenocytes from OT-1 mice [C57BL/6-Tg(TcraTcrb)1100Mjb/J] were processed, and CD8^+^ T cell effectors (OT-1 cells) were isolated using a MACS CD8a^+^ T cell isolation kit (Miltenyi). Targets consisted of DC2.4 mouse (H2K^b^) dendritic cells that were either noninfected (control) or infected (MOI, 100) overnight with Brucella (B. melitensis, B. melitensis ova-cfp 3, B. melitensis ova-cfp 4). Positive control consisted of cells pulsed with the OVA peptide (SIINFEKL) as described above. Targets, effectors, and controls were plated, incubated, and assayed as described for the immune effector CTL assay above, using the manufacturer's (Promega) recommended protocol and calculations for percent specific cytotoxicity.

### IFN-γ ELISA.

Effector cells were prepared by immunizing mice (C57/BL6) as described for the CTL experimental group above, except that an additional group was immunized with the OVA peptide (SIINFEKL) at 50 μg in 0.2 ml Sigma adjuvant system (Sigma) i.p. according to the manufacturer's protocol. Peptide immunizations were boosted after 2 weeks, and 1 week later, splenocytes were harvested. Splenocytes from each animal were then stimulated in culture with 1 μg/ml of SIINFEKL peptide and incubated for 48 h at 37°C. Cultured supernatants were harvested, and IFN-γ amounts were determined using the ELISA Ready-Set-Go! system (Affymetrix; eBioscience) according to the manufacturer's protocol.

### β-Galactosidase X-Gal and ONPG assays.

Cultures containing a mixture of B3Z T cell hybrids and DC2.4 APCs (2 × 10^5^ cells/ml each) were plated in 6-well tissue culture plates (X-Gal assays) or 96-well flat-bottom tissue culture plates (ONPG assays), and peptides or bacteria were added. Peptides (listed in Table 2) were synthesized and purchased from GenScript and resuspended in dimethyl sulfoxide (DMSO) at a stock concentration of 20 mg/ml. B. melitensis and variants were used at an MOI of 100. For some assays, APCs were treated with tauroursodeoxycholic acid (TUDCA; Sigma) at 100 μg/ml, or tunicamycin (Sigma) at 10 μg/ml, or mouse IFN-γ (PromoKine) at 1 μg/ml. After overnight incubation, cells were washed in PBS and fixed (X-Gal assay) or lysed (ONPG assay). For X-Gal staining, cells were fixed with 4% paraformaldehyde (PFA) for 10 min, washed 3 timed in PBS, and overlaid with a solution of 1 mg X-Gal/ml, 5 mM potassium ferrocyanide, 5 mM potassium ferricyanide, and 2 mM MgCl_2_. After an overnight incubation at 37°C, plates were examined microscopically for the presence of blue (lacZ expressing) cells. For ONPG staining, we used a SensoLyte ONPG β-galactosidase assay kit (AnaSpec, Inc.) as per the manufacturer's recommended protocol, except that the incubation at 37°C was overnight. Absorbance reading was at 405 nm.

### Prediction of near neighbors.

Structural diagrams of binding of SIINFEKL in the H2K^b^ murine MHC-I molecule (3P9L) (61) illustrate that this octamer is bound with its C-terminal leucine located in the P9 pocket position and the N-terminal serine in the P2 pocket position. This results in a pentamer peptide exposed to the T cell receptors (T cell-exposed motif [TCEM]) (41). Amino acids N, E, and K protrude particularly prominently from the MHC groove in positions P5, P7, and P8. By replacing amino acids with those of similar physicochemical characteristics in T cell-exposed positions P4 to P8, 31 peptides that a T cell receptor would likely tolerate and bind as an alternate “near neighbor” of SIINFEKL were identified. The amino acid substitutions in the exposed positions included I changed to L in P4, N to Q in P5, F to I, A, L, or Y in P6, E to D in P7, and K to R in P8. The B. melitensis proteome and the proteomes of an array of other pathogenic and microbiome bacteria were then searched to determine the occurrence of each of the alternate TCEM pentamer motifs P4 to P8. In places where a near neighbor was identified in B. melitensis, the flanking amino acids were noted, the predicted binding to murine MHC-I alleles was determined in the context of the native Brucella protein using previously described methods (62), and the probability of cathepsin cleavage at the C terminus of that peptide was determined (23, 63).

### Statistical analyses.

Analysis of variance (ANOVA) was used to analyze the differences among group means. Tukey's honestly significant difference (HSD) test was used as the *post hoc* follow-up test comparing every group mean with every other group mean to determine significant differences among groups.

## Supplementary Material

Supplemental material
