# Supplementary material for: Brucella Peptide Cross-Reactive Major Histocompatibility Complex Class I Presentation Activates SIINFEKL-Specific T Cell Receptor-Expressing T Cells
Source: Infect Immun. 2018 Jun 21;86(7):e00281-18. doi: 10.1128/IAI.00281-18 (PMC6013681; doi:10.1128/IAI.00281-18)
Supplement: Supplemental material [file supp_86_7_e00281-18__index.html]

Supplemental material 

# Brucella Peptide Cross-Reactive Major Histocompatibility Complex Class I Presentation Activates SIINFEKL-Specific T Cell Receptor-Expressing T Cells

## Supplemental material

- Supplemental file 1 -

  Fig. S1. Plasmid map of pMOD3-OVA-ECFP generated by SnapGene software (GSL Biotech).

  PDF, 298K
- Supplemental file 2 -

  Table S1. *Brucella* proteins with SIINFEKL near-neighbor attributes.

  PDF, 58K
- Supplemental file 3 -

  Table S2. Frequencies of near-neighbor peptides in bacterial pathogens.

  PDF, 280K
