# Supplementary figures and images for: Brucella Peptide Cross-Reactive Major Histocompatibility Complex Class I Presentation Activates SIINFEKL-Specific T Cell Receptor-Expressing T Cells
Source: Infect Immun. 2018 Jun 21;86(7):e00281-18. doi: 10.1128/IAI.00281-18 (PMC6013681; doi:10.1128/IAI.00281-18)

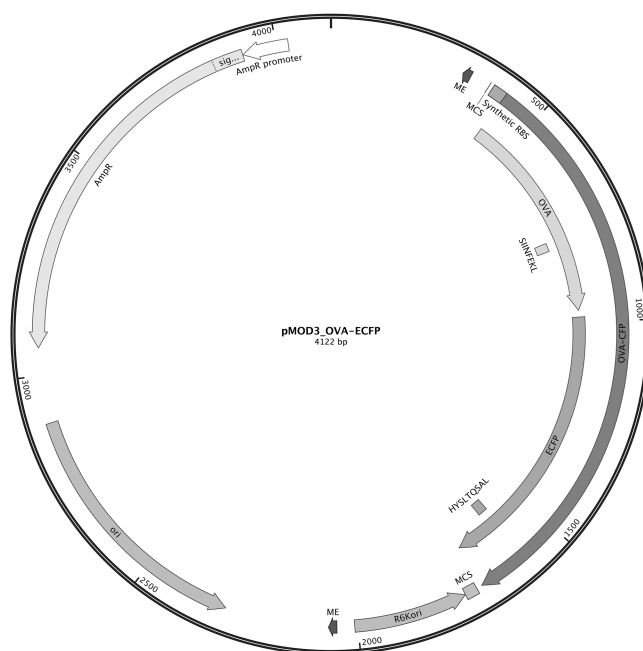

Supplement: Supplemental material [file IAI.00281-18_zii999092461s1.pdf]
