# Supplementary material for: Brucella Peptide Cross-Reactive Major Histocompatibility Complex Class I Presentation Activates SIINFEKL-Specific T Cell Receptor-Expressing T Cells
Source: Infect Immun. 2018 Jun 21;86(7):e00281-18. doi: 10.1128/IAI.00281-18 (PMC6013681; doi:10.1128/IAI.00281-18)
Supplement: Supplemental material [file IAI.00281-18_zii999092461st2.pdf]

Supplemental Table 1. *Brucella* proteins with SIINFEKL near neighbor attributes.

gI number[1]	P4-P8 TCEM[2]	9-mer	Curation	Index amino acid	H2K[b3]	H2D[b]	Cathepsin excision[4]
17853629	~~~LQIDK~	AAYLQIDKI	FIG00450714_ hypothetical protein	27	-2.00	-2.26	0
17852629	~~~INADK~	VIIINADKV	LSU ribosomal protein L13p (L13Ae)	54	-1.84	-2.07	10
17855512	~~~LQLDK~	KPYLQLDKI	Dipeptidyl carboxypeptidase Dcp (EC 3_4_15_5)	360	-1.45	-2.14	10
17855925	~~~IQFEK~	SSSIQFEKV	Aldehyde dehydrogenase A	279	-1.35	-1.59	10
17855142	~~~INLER~	NAFINLERG	Nucleoside-diphosphate-sugar epimerases	164	-1.20	-2.1	0
17851235	~~~LQLDR~	GLRLQLDRM	Transcriptional regulator	3	-1.19	-0.87	10
17849989	~~~INLDK~	ATDINLDKV	FIG00450547_ hypothetical protein	33	-1.18	-1.05	10
17856679	~~~LNLDK~	GMVLNLDKC	Respiratory nitrate reductase beta chain	8	-1.18	-1.51	10
17853294	~~~INAER~	KSIINAERL	Guanylate kinase	175	-1.17	-2.56	9
17854371	~~~INLDK~	KNKINLDKL	Translation initiation factor 2	604	-0.94	-1.46	10
17850913	~~~INLDK~	CYLINLDKS	Glycosyltransferase	3	-0.81	0.19	0
17852677	~~~INLER~	GRKINLERS	Riboflavin synthase alpha chain	83	-0.56	1.32	0
17853930	~~~LNIER~	LRLLNIERA	Methylated-DNA--protein-cysteine methyltransferase	162	-0.48	-0.56	9
17851725	~~~INYDK~	FPEINYDKV	LSU ribosomal protein L5p (L11e)	144	-0.47	-1.16	0
17849941	~~~INLDR~	YPDINLDRL	tRNA uridine 5-carboxymethylaminomethyl modification enzyme GidA	513	-0.40	-1.04	10
17852543	~~~INAER~	EADINAERF	Inositol monophosphatase and related sulfite synthesis enzyme	47	-0.23	-1.74	10
17853830	~~~LQAER~	SYRLQAERA	Purine_pyrimidine phosphoribosyl transferase	34	-0.17	0.96	ND
17851993	~~~IQADK~	VYRIQADKE	Transcriptional regulator	92	-0.10	1.53	0
17855333	~~~LNIER~	KHGLNIERI	Methionine ABC transporter substrate-binding protein	64	-0.08	-1.08	0
17851583	~~~LQAEK~	LAMLQAEKA	Cobalamin biosynthesis protein CobG	128	0.04	-1.47	10
17851317	~~~INFDK~	KLTINFDKA	ATP-dependent DNA helicase UvrD_PcrA	837	0.23	-0.62	0
17854960	~~~INIDR~	PQKINIDRT	Zinc ABC transporter	70	0.24	0.38	11
17851851	~~~LNLER~	KERLNLERE	SSU ribosomal protein S2p (SAe)	133	0.31	-0.27	ND
17851168	~~~LNADK~	LVYLNADKD	Sodium-dependent phosphate transporter	93	0.40	-0.13	ND
17855226	~~~LQAEK~	GINLQAEKD	L-2-hydroxyglutarate oxidase	396	0.60	0.52	ND
17853130	~~~LQIDR~	VRGLQIDRN	D-xylose transport ATP-binding protein XylG	372	0.70	1.16	ND
17854502	~~~LQWER~	PQFLQWERE	FIG041266_ ATP-dependent nuclease subunit B	866	0.78	-0.36	ND
17853010	~~~LNAEK~	RRILNAEKD	Cytochrome c heme lyase subunit CcmH	70	0.89	-0.05	ND
17851529	~~~LQWDR~	PQALQWDRD	Branched-chain acyl-CoA dehydrogenase	32	0.91	1.03	ND
17853996	~~~LQLDK~	NGRLQLDKD	FIG00793979_ hypothetical protein	164	0.99	0.49	ND
17850592	~~~LNFDR~	KKALNFDRD	FIG00450278_ hypothetical protein	21	1.00	0.03	ND
17850456	~~~LQADK~	KSALQADKA	NAD-specific glutamate dehydrogenase	1444	1.01	-0.88	0
17850175	~~~INLEK~	RQIINLEKR	Transcriptional regulator	41	1.59	-0.40	ND
17850438	~~~INADR~	QATINADRN	Membrane-bound lytic murein transglycosylase B precursor	70	1.67	-0.91	0

17853637	~~~IQLDR~	QYDIQLDRE	RND efflux system	722	1.76	0.23	ND
17853483	~~~IQIER~	DITIQIERD	Iron-responsive regulator Irr	181	1.84	0.32	ND
17856600	~~~LNIDR~	QLNLNIDRQ	RND efflux system	327	2.16	0.25	ND
17850726	~~~LQAER~	MMGLQAERD	Hypothetical protein	113	2.38	0.43	ND

[1]gi number can be converted using ID mapping tool at UniProt (http://www.uniprot.org/help/uploadlists).

[2]P4-P8 TCEM indicates the pentamer exposed to the T cell receptor when a nonamer is bound in a MHC I; amino acids in hidden anchor positions are represented by ~.

[3]Binding affinity for $H2K^b$ and $H2D^b$ is shown in standard deviation units relative to the mean for the protein of origin. More negative numbers indicate higher probability of binding.

[4]Cathepsin excision: probability over 50% for cathepsin S or L is shown as 9,10, or 11 amino acid peptide; 0 indicates no predicted excision. ND indicates not determined (for proteins with low binding probability).
