# Supplementary material for: Brucella Peptide Cross-Reactive Major Histocompatibility Complex Class I Presentation Activates SIINFEKL-Specific T Cell Receptor-Expressing T Cells
Source: Infect Immun. 2018 Jun 21;86(7):e00281-18. doi: 10.1128/IAI.00281-18 (PMC6013681; doi:10.1128/IAI.00281-18)
Supplement: Supplemental material [file IAI.00281-18_zii999092461st3.pdf]

Supplemental Table 2. Frequency of near neighbor peptides in bacterial pathogens

	P4-P8 TCEM Motif																														
Bacterial Strains	INADK	INADR	INAER	INFDK	INFEK	INIDR	INLDK	INLDR	INLEK	INLER	INYDK	IQADK	IQFEK	IQIER	IQLDR	LNADK	LNAEK	LNFDR	LNIDR	LNIER	LNLDK	LNLER	LQADK	LQAEK	LQAER	LQIDK	LQIDR	LQLDK	LQLDR	LQWDR	LQWER	Total Near Neigbors
Bordetella_bronchiseptica_2371640								1	1			1					1	1			4	3			3	1			1			17
Bordetella_bronchiseptica_253			1					1	1			1					1	1	1			6			4	1			1			19
Bordetella_bronchiseptica_D445			1					1	1			1					1	1	1			5	1		5	1						19
Bordetella_bronchiseptica_RB50			1					1	1			1					1	1	1			6			4	1			1			19
Bordetella_bronchiseptica_SEAT0006								1	1			1					1	1			12	3			3	1			1			25
Bordetella_pertussis_B1920								1	1			1					1	1				3			3	1			1			13
Bordetella_pertussis_Bp_H897								1	1			1					1	1			13	3			3	1			2			27
Bordetella_pertussis_Bp_SEAT_0004								1	1			1					1	1				3			3	1			1			13
Bordetella_pertussis_CHLA15								1	1			1					1	1			1	3			3	1			1			14
Bordetella_pertussis_Tohama_I								1	1			1					1	1			14	3			3	1			1			27
Brucella_melitensis_043	1		2	1		1	3	1	1	2	1	1	1	1	1	1			1	2	2	1	1	2	2	1	1	2	1	1	1	36
Brucella_melitensis_ATCC_23457	1	1	2	1		1	2	1	1	2	1	1	1	1	1	1	1	1	1	2	1	1	1	2	2	1	1	2	1	1	1	37
Brucella_melitensis_B115	1	1	2	1		1	2	1	1	2	1	1	1	1	1	1	1		1	2	1	1	1	2	2	1	1	2	1	1	1	36
Brucella_melitensis_M5	1	1	2	1		1	3	1	1	2	1	1	1	1	1	1	1		1	2	1	1	1	2	2	1	1	2	1	1	1	37
Brucella_melitensis_S66	1	1	2	1		1	3	1	1	2	1	1	1	1	1	1	1		1	2	1	1	1	2	2	1	1	2	1		1	36
Burkholderia_cenocepacia_AU_1054		1		1	1	1			3					1	1		2					3	1	1	4	1	2	2	2			27
Burkholderia_cenocepacia_H111		1			1	1			3		1			3	1		2					2	1	1	3	1	1		3			25
Burkholderia_cenocepacia_KC-01		1			1	1			2								2					1			5	1	1	1	1			17
Burkholderia_cepacia_ATCC_25416		2			1	1	2		2					1	1		4			1	1	3			2	1	2	2	3			29
Burkholderia_cepacia_Bu72		1			1	1			2	1				1	1		2				1	4	1	2	4	1	1		1			25
Burkholderia_mallei_ATCC_10399						1			2							1	1				2	2			1	1	1		3			15
Burkholderia_mallei_ATCC_23344						1			2							1	1				2	2			1	1	1		2			14
Burkholderia_mallei_FMH						1			2							1	1				2	2			1	1	1		2			14
Burkholderia_mallei_JHU						1			2							1	1				1	2			1	1	1		3			14
Burkholderia_mallei_NCTC_10247						1			2							1	1				2	2			1	1	1		2			14
Burkholderia_pseudomallei_1026b	1					1	1		2							1	1				3	2		1	1	2	1		2			19
Burkholderia_pseudomallei_1106a	1	1				1	1		2							1	1				3	2		1	1	1	1		2			19
Burkholderia_pseudomallei_1106b	1	1				1	1		2							1	1				3	2		1	1	1	1		2			19
Burkholderia_pseudomallei_K96243	1					1			2							1	1	1			3	2		1	1	2	1		2			19

Burkholderia_pseudomallei_MSHR305	1					1	1		2						1	1	1	1			3	2		1	1	1	1		2			20
Chlamydia_trachomatis_A363			1													1					1			1								4
Chlamydia_trachomatis_Ds2923			1													1					1			1								4
Chlamydia_trachomatis_FSW5			1													1					1			1								4
Chlamydia_trachomatis_Jali20			1													1					1			1								4
Chlamydia_trachomatis_Sweden2			1													1					1			1								4
Clostridium_difficile_ATCC_43255			2	5	2	1	5	2	3	1	2		1			1	1	2	2		2	2		1		2		2				39
Clostridium_difficile_CD165			2	5	2		6	2	3		2		1			1	1	2	4		2	1		2		1		3				40
Clostridium_difficile_DA00212			2	5	1		6	2	4	1	2		1			1	1	2	3		2	1				1		3				38
Clostridium_difficile_Y270			2	4	2		5	2	1	1	2		1			1	1	2	3		2	1				1		3				34
Clostridium_perfringens_ATCC_13124	1			2	1	1	6	1	4		1	1		1						2	5							1				27
Clostridium_perfringens_CPE_str_F4969	1			2	2	1	5	2	3		1	1		1						3	5							1				28
Clostridium_perfringens_E_str_JGS1987	1			3	3	2	4	1	6		2	1		1			1			4	4	2	1					1				37
Clostridium_perfringens_JJC	1			2	1	1	5	2	5		1	1		1						2	4	1										27
Clostridium_perfringens_str_13	1			2	1	1	4	1	3	1	1	1							1	2	6	2										27
Coxiella_burnetii_Cb185	1					1		1	3	3			1	1	2		1				1	2		2	2							21
Coxiella_burnetii_Dugway_5J108-111	1					1		1	3	3	1		2	2	2		1					2	1	2	1							23
Coxiella_burnetii_RSA_331	1					1		1	3	2	1		1	1	2		1				1	2		1	1							19
Coxiella_burnetii_RSA_493	1					1		1	3	2	1		1	1	2		1				1	2		2	1							20
Coxiella_burnetii_Z3055	1					1		2	3	2	1		1	1	2						1	2		2	2							21
Francisella_novicida_FTE	1			1		1	2	2	2							1					4			2				1				17
Francisella_novicida_FTG	1			1		1	2	2	2							1					4			2		1		1				18
Francisella_novicida_GA99-3548	1			1	1	1	2	2	1							1					4			2								16
Francisella_novicida_GA99-3549	1			1		1	3	2	1							2		1			4			2				1				19
Francisella_novicida_U112	1			1		1	2	2	2					1		1					4			2				1				18
Francisella_tularensis_subsp_holarctica	1			1		1	2	1	1					1		1					4			2								15
Francisella_tularensis_subsp_holarctica_257	1			1		1	2	1	1							1					3			2								13
Francisella_tularensis_subsp_holarctica_F92	1			1		1	2	1	1					1		1					4			2								15
Francisella_tularensis_subsp_holarctica_FSC022	1			1		1	2	2	2					1		1					3			2								16
Francisella_tularensis_subsp_holarctica_FSC200	1			1		1	2	1	1					1		1					4			2								15

Francisella_tularensis_subsp_tularensis_FSC033	1			1		1	3	2	2							1					3			3								17
Francisella_tularensis_subsp_tularensis_FSC198	1			1		1	3	2	2					1		1					4			3								19
Francisella_tularensis_subsp_tularensis_MA00-2987	1			1		1	3	2	2							1					4			3								18
Francisella_tularensis_subsp_tularensis_NE061598	1			1		1	3	2	2					1		1					4			3								19
Francisella_tularensis_subsp_tularensis_SCHU_S4	1			1		1	3	2	2					1		1					4			3								19
Listeria monocytogenes EGD	2		4	0	1		3		2	2	1	1				3	3			2	0	0	1	2	2	1		0	1			31
Listeria monocytogenes EGD-e	2		4	0	1		3		3	2	1	1				3	3			2	0	0	1	2	2	1		0	1			32
Listeria monocytogenes strain CFSAN023459	2		2	1	0		3		1	2	2	1				2	2			0	1	1	1	2	2	1		2	1			29
Listeria monocytogenes strain Lm 3136	2		4	0	1		3		2	2	1	1				3	3			2	0	0	1	2	2	1		2	1			33
Mycobacterium_abscessus_4S-0303		1	1			1													2	1	1	1		1	1						3	13
Mycobacterium_abscessus_5S-1212		1	1			1		1				1			1				2		1			1	2						2	14
Mycobacterium_abscessus_6G-0212		1	1			1				1				1					2	1	1	1		1	1						3	15
Mycobacterium_abscessus_M156		2	2			1						1			1				2	1	1			1	1						2	15
Mycobacterium_abscessus_V06705	1	1	1			1				1				1			1		2	1	1	1		2	1						3	18
Mycobacterium_bovis_BCG_str_ATCC_35733	1	2	2					2							2	1					1			1	3				2		2	19
Mycobacterium_bovis_BCG_str_ATCC_35740	1	2	2					2							2	1					1			1	3				2		2	19
Mycobacterium_bovis_BCG_str_ATCC_35743	1	2	2					2							2	1					1			1	3				2		2	19
Mycobacterium_bovis_BCG_str_Glaxo	1	2	2					2							2	1					1			1	3				2		2	19
Mycobacterium_bovis_BCG_str_Pasteur_1173P2	1	2	2					2							1	1					1			1	3				2		2	18
Mycobacterium_leprae_Br4923	2	1		1											2										2				1			9
Mycobacterium_leprae_TN	2	1		1											2										2				1			9
Mycobacterium_tuberculosis_H37Ra	1	2	2					2							1	1					1			1	3				2		2	18
Mycobacterium_tuberculosis_H37RvAE	1	2	2					2							2	1					1			1	3				2		2	19
Mycobacterium_tuberculosis_H37RvCO	1	2	2					2							2	1					1			1	3				2		2	19
Mycobacterium_tuberculosis_H37RvHA	1	2	2					2							2	1					1			1	3				2		2	19
Mycobacterium_tuberculosis_H37RvJO	1	2	2					2							2	1					1			1	3				2		2	19
Neisseria_gonorrhoeae_1291	1						2		1	1		1					2	1		1	1			2	3				1			17
Neisseria_gonorrhoeae_DGI2	1						2		1	1		1					2	1		1	1			2	3				1			17
Neisseria_gonorrhoeae_FA_1090	1						2		1	1		2					2	1		1	1			2	2				1			17
Neisseria_gonorrhoeae_PID18	1						2		1	1		1					2	1		1	1			2	3				1			17

Neisseria_gonorrhoeae_SK-93-1035	1						2	1	1	1		1					2	1		1	1			2	3				1			18
Neisseria_meningitidis_2003022	2				1				1	1	1						2			2	1	1		3	4		1		1			21
Neisseria_meningitidis_2004032	1								1	1	1					1	2			2	1	1		2	4				1			18
Neisseria_meningitidis_64182	2				1				1	1	1						2			1	1	1		3	4		1					19
Neisseria_meningitidis_93003	1								1	1	1	1					2			1	1	1		2	3				1			16
Neisseria_meningitidis_NM3222	1								1	1	1						2			1	1	1		2	4				1			16
Salmonella enterica subsp. enterica serovar Typhimurium str. 14028S	1	1	2		1		9	1	2		1				1	3		1			3	3	0	6	4			4	1			44
Salmonella enterica subsp. enterica serovar Typhimurium str. CDC 2009K-2059	1	1	2		1		9	1	2		1				1	3		1			3	3	1	6	4			4	1			45
Salmonella enterica subsp. enterica serovar Typhimurium strain 22792	1	1	2		1		9	1	2		1				1	3		1			3	3	0	6	4			4	1			44
Salmonella enterica subsp. enterica serovar Typhimurium strain ATCC 13311	1	1	2		1		9	1	2		1				1	3		1			2	3	0	6	4			4	1			43
Staphylococcus_aureus_subsp_aureus_ATCC_51811	1				3	1	1		3		2			2				1	4	5	1	3			1	1						29
Staphylococcus_aureus_subsp_aureus_ATCC_BAA-39	1				3	1	1		3		2			2		1		1	4	5		1			1	1						27
Staphylococcus_aureus_subsp_aureus_COL	1				3	1	1		3		2			2				1	4	4	1	1			1	1						26
Staphylococcus_aureus_subsp_aureus_MRSA131	1				2	1	1		2		1			2		1		1	4	5		2			1	1						25
Staphylococcus_aureus_subsp_aureus_USA300_TCH1516	1				3	1	1		3		2			2		1		1	4	5		1			1	1						27
Staphylococcus_epidermidis_NIH051668	2				2						1								3	2	1							1				12
Staphylococcus_epidermidis_VCU120	2				2				1		1								3	2	1							1				13
Staphylococcus_epidermidis_VCU139	1			1		1		2	1		1			2					2	4	1											16
Staphylococcus_epidermidis_W23144	2				1						1								3	2	1											10
Staphylococcus_epidermidis_WI05	1				2						1								4	2	1											11
Streptococcus_agalactiae_A909	1			1		1	2	1	1		2		1			2				2	1	1		3				1	1			21
Streptococcus_agalactiae_CCUG_24810	1			1		1	2	1	1		2		1			3				2				3		1		1	1			21
Streptococcus_agalactiae_GB00247	1			1		1	2	1	1		2		1			3				2	1			3				1				20
Streptococcus_agalactiae_GB00951	1			1		1	2	1	1		2		1			3				2				3		1		1				20
Streptococcus_agalactiae_ILRI005	1			1		1	2	1	1		2		1			1				1				3		1		1				17
Streptococcus_dysgalactiae_subsp_dysgalactiae_ATCC_27957	1	1			1		2	1	1		1													2				1				11
Streptococcus_dysgalactiae_subsp_equisimilis_167	1				1		1		2		1													1								7
Streptococcus_dysgalactiae_subsp_equisimilis_ATCC_12394	1				1		2		1		1						1				1			1								9

Streptococcus_dysgalactiae_subsp_equisimilis_GGS_124	1				1		1	1	1		1					1					1			1					1			10
Streptococcus_dysgalactiae_subsp_equisimilis_RE378	1						1		1		1					1	1				1			1								8
Streptococcus_mutans_TCI-110	1		1		1			1			1					2	2				1	1					1					12
Streptococcus_mutans_TCI-149	1		1		1			1			1					2	2				1	1					1					12
Streptococcus_mutans_TCI-223	1		1		1			1			1					2	2				1	1					1					12
Streptococcus_mutans_TCI-400	1		1		2	1		1	1		1					2	2				1	1					1					15
Streptococcus_mutans_TCI-92	1		1		2	1		1	2		1					2	2				1	1					1					16
Streptococcus_pneumoniae_ATCC_700669				2			1	1	2	1			1				1				2		1	2					1			15
Streptococcus_pneumoniae_Hungary19A-6				2		1	1	1	1	1			1				1				2		1	2								14
Streptococcus_pneumoniae_SP195				2		1	1	1					1				1				2		1	3					1			14
Streptococcus_pneumoniae_Taiwan19F-14				2		1	1	1	1				1	1			1				2		1	1								13
Streptococcus_pneumoniae_TCH843119A				2		1	1	1	1				1	1			1				2		1	1								13
Streptococcus_pyogenes_GA03805	1				1		1	1	3		1						1				4			2								15
Streptococcus_pyogenes_GA16797	1				1		1		3		1						1				4			3					1			16
Streptococcus_pyogenes_GA19681	2				1		1	1	2		1						1				4			2								15
Streptococcus_pyogenes_MGAS2096	1				1		1		3		1						1				4			2					1			15
Streptococcus_pyogenes_UTMEM-1	1				1		1		4		1						1				4			2				1				16
Ureaplasma_urealyticum_2608			1		1				3		1		1				1						1		3							12
Ureaplasma_urealyticum_serovar_11_str_ATCC_33695			1		1				3		1		1				1						1		2							11
Ureaplasma_urealyticum_serovar_2_str_ATCC_27814			1		1				3		1		1				1						1		2							11
Ureaplasma_urealyticum_serovar_7_str_ATCC_27819			1		1				3		1		1				1						1		2							11
Ureaplasma_urealyticum_serovar_9_str_ATCC_33175			1		1				3		1		1				1						1		3							12
